# Differences of rhizospheric and endophytic bacteria are recruited by different watermelon phenotypes relating to rind colors formation

**DOI:** 10.1038/s41598-022-10533-0

**Published:** 2022-04-15

**Authors:** Jian Xiao, Si-yu Chen, Yan Sun, Shang-dong Yang, Yi He

**Affiliations:** 1 Guangxi Key Laboratory of Subtropical Bio-resource Conservation and Utilization, Agricultural College, Guangxi University, Nanning, 530004 Guangxi People’s Republic of China; 2 Horticultural Research Institute, Guangxi Academy of Agricultural Sciences, Nanning, 530007 Guangxi People’s Republic of China

**Keywords:** Microbiology, Plant sciences

## Abstract

To elucidate the biological mechanism of yellow rind formation on watermelon, the characteristics of soil bacterial community structure in rhizosphere and endophytic bacteria in stem of yellow rind watermelon were analyzed. Based on high-throughput sequencing technology, plant stem and rhizosphere soil samples, which collected from yellow and green rind watermelons were used in this paper, respectively. The structural characteristics of the endophytic bacteria in stems and soil bacterial communities in rhizospheres of yellow and green rind watermelons were comparative studied. Firstly, significant different proportions of some dominant bacteria and abundances could be detected between yellow and rind watermelons. Meanwhile, although different abundances of endophytic bacteria could be found, but no significant differences were observed between yellow and green rind watermelons. Moreover, Gemmatimonadota, Myxococcota, WPS-2, *norank_f_Gemmatimonadaceae* and *Bradyrhizobium* were the soil dominant bacterial genera in rhizosphere of green rind watermelon. All above results suggest that differences of rhizospheric and endophytic bacteria are exactly recruited as “workers” by different watermelon phenotypes relating to rind color formations.

## Introduction

Watermelon [*Citrullus lanatus* (Thunb.) Matsum. and Nakai var. *lanatus* (2n = 2x = 22)] is a Cucurbitaceae family horticulture crop that is also one of the top ten most eaten fresh fruits on the planet^1^. Watermelons have become one of the model crops for fruit-quality study due to their wide range of characteristics, including form, size, rind thickness and color, flesh texture and color, and sugar and carotenoids content^1,2^.

Watermelon exhibits a wide range of rind colors: dark or light green, light green-gray, and yellow are the common colors^3–5^. The rind color of watermelon is an important commodity in modern society for its gorgeous appearance is significance improving the quality, which is considered as an important trait for influencing consumer preference and focusing of breeders^6,7^.

The key pigments that impact watermelon rind colors are chlorophyll and carotenoids, which play crucial roles in gathering light energy and converting it to chemical energy^1^. Chlorophyll plays a crucial role in photosynthesis, as everyone knows^8^. Carotenoids are 40-carbon isoprenoids that serve important functions in photosynthesis and photoprotection in photosynthetic organisms, as well as providing evolutionarily adaptive colorations in plants, fungi, and animals^9,10^. Various taxa, including bacteria, archaea, fungus, algae, land plants, and mammals, have been shown to contain over 750 structurally distinct carotenoids^11^.

At present, studies have also confirmed that soil microbes in rhizospheres of crops contribute to sustainable crop production^12–14^, such as the mitigation of environmental stresses, drought and cold or heat damages, etc.^15–17^. Endophytic bacteria are distributed in all internal parts of the plant and have functions in nitrogen fixation^18,19^, phosphorus solubilization^20,21^, production of plant growth regulating substances^22,23^, enhancement of host resistance^24,25^ and bioremediation^26^, which play an important role in regulating the microecological balance for enhancing resistance and promoting the healthy growth of the host plant^27^.

Studies also have found that endophytic bacteria are closely associated with the production of plant endogenous hormones, such as growth auxin^28^, cytokinin^29^, abscisic acid^30^, gibberellin^31^, and ethylene^32^. Endophytic bacteria also could increase the chlorophyll content in tomato^33^ and *P. edulis*^34^.

Recently, watermelon with yellow rind has gained increasing popularity among consumers^35^. However, the genes how to regulate forming yellow or green rind and their molecular mechanisms are still unknown in watermelon. And whether the rhizospheric and endophytic bacteria contributes to rind color formation or not are also still unclear. In this study, to explore the compositions and functions of the rhizospheric and endophytic bacteria whether relates to rind color formation of watermelon differences of rhizospheric and endophytic bacterial compositions between yellow and green rinds watermelon were comparative analyzed.

## Results

### Overall structure of rhizospheric and endophytic bacterial compositions across samples

**Figure 1:**
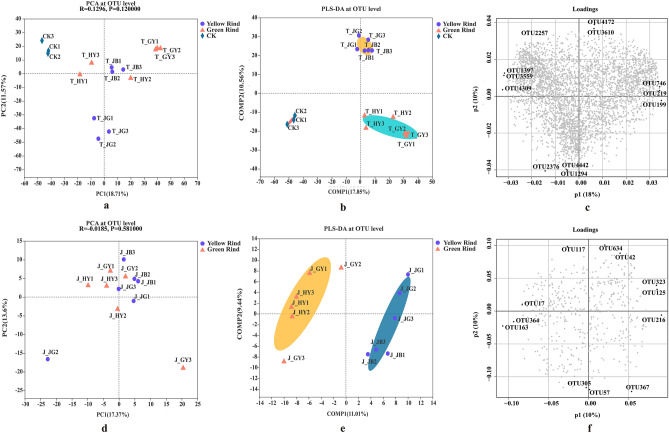
Comparison of soil bacteria in rhizosphere and endophytic bacteria between yellow and green rinds of watermelons. (**a**) PCA of soil bacteria communities at the OTU level. (**b**) PLS-DA score plot of soil bacteria communities in rhizosphere between yellow and green rinds of watermelons. (**c**) PLS-DA loading plot of soil bacterial communities in rhizosphere between yellow and green rinds of watermelons. (**d**) PCA of endophytic bacteria communities at the OTU level. (**e**) PLS-DA score plot of endophytic bacteria communities between yellow and green rinds of watermelons. (**f**) PLS-DA loading plot of endophytic bacterial communities between yellow and green rinds of watermelons.

To evaluate the extent of the similarity of the rhizospheric and endophytic bacterial communities, unweighted UniFrac Principal Component Analysis (PCA) at OTU level was performed. The results suggested that soil bacterial compositions in rhizosphere of yellow and green rinds of watermelons were quite significant differences with the CK (Fig. 1a), but it was quite similarity with the endophytic bacterial compositions between yellow and green rinds of watermelons (Fig. 1d). Meanwhile, Partial Least Squares Discriminant Analysis (PLS-DA), a supervised analysis suitable for high-dimensional data was also carried out (Fig. 1b,c,e,f). All above results suggested that the rhizospheric and endophytic bacterial communities between yellow and green rind of watermelons clustered separately, it suggested that the rhizospheric and endophytic bacterial community structures between yellow and green rind of watermelons were significantly different.

### Common and distinct bacterial taxa in the analyzed samples

**Figure 2:**
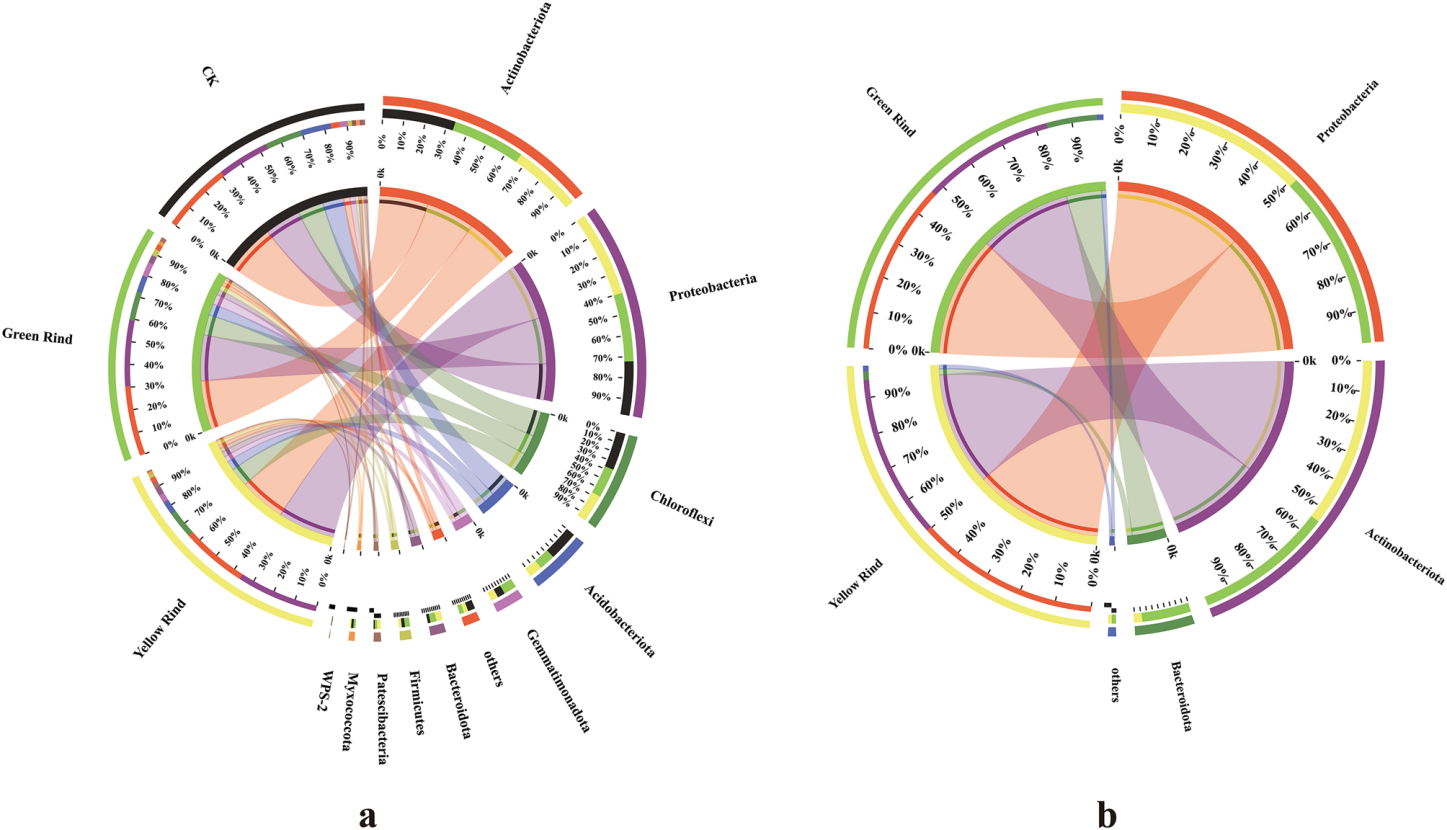
Distribution of soil dominant bacteria in rhizosphere (**a**) and endophytic (**b**) bacteria between yellow and green rinds of watermelons at phylum level. The data were visualized by Circos. The width of the bars from each phylum indicate the relative abundance of that phylum in the sample. The small half-circle (left half-circle) represents the species composition in the sample, the color of the outer colored band represents the grouping from which the species is derived, the color of the inner colored band represents the species, and the length represents the relative abundance of the species in the corresponding sample. The large half-circle (right half-circle) represents the proportional distribution of species in different samples at phylum level, the outer colored band represents the species, the inner the color of the colored band represents different groups, and the length represents the proportion of distribution of the sample in a particular species.

The proportions and distribution of rhizospheric soil and endophytic dominant bacterial compositions of different samples can be reflected by Circos plots^36^ (Fig. 2). At the phylum level, soil dominant bacteria in rhizosphere, which its proportion is greater than 1% among yellow, green rind watermelons and CK are shown in Fig. 2a. The dominant bacterial phyla of CK from high to low were Actinobacteriota (31.41%), Proteobacteria (23.17%), Chloroflexi (16.47%), Acidobacteriota (13.57%), Gemmatimonadota (3.70%), Firmicutes (1.89%), WPS-2 (1.84%), Myxococcota (1.69%), Bacteroidota (1.45%) and others (3.96%), respectively. And the soil dominant bacterial phyla in rhizosphere of yellow rind watermelon were Proteobacteria (36.55%), Actinobacteriota (29.66%), Chloroflexi (12.24%), Acidobacteriota (6.02%), Gemmatimonadota (3.63%), Bacteroidota (3.48%), Patescibacteria (2.62%), Firmicutes (1.81%), Myxococcota (1.16%) and others (2.42%), respectively. By contrast, soil dominant bacterial phyla in rhizosphere of green rind watermelon were Actinobacteriota (31.14%), Proteobacteria (29.50%), Chloroflexi (13.11%), Acidobacteriota (7.34%), Gemmatimonadota (6.43%), Bacteroidota (3.47%), Firmicutes (3.10%), Myxococcota (1.72%), Patescibacteria (1.64%) and others (2.40%), respectively. i.e. Actinobacteriota, Proteobacteria, Chloroflexi, Acidobacteriota and Gemmatimonadota were not only the five most dominant soil bacterial phyla of CK, but also were the five most dominant soil bacterial phyla in rhizosphere of yellow and green rinds of watermelons too. However, the proportions of the above five soil dominant bacterial phyla in CK were significantly altered in rhizospheres of yellow and green rinds watermelons. Moreover, the proportions of soil dominant bacterial phyla in rhizosphere between yellow and green rinds watermelons were also significantly changed in accordance with the varieties of watermelons. Among them, Proteobacteria was the most abundant soil bacterial phylum in rhizosphere of yellow rind watermelon, and Gemmatimonadota was the most abundant soil bacterial phylum in rhizosphere of green rind watermelon.

In addition, the compositions of the dominant endophytic bacteria in stems of yellow rind watermelon were Proteobacteria (51.03%), Actinobacteriota (45.07%), Bacteroidota (2.36%) and others (1.54%), respectively. By contrast, Proteobacteria (47.61%), Actinobacteriota (36.70%), Bacteroidota (13.88%) and others (1.82%) were also the dominant endophytic bacteria in stems of green rind watermelons. That is, the proportions of dominant endophytic bacterial phyla in stems between yellow and green rinds watermelon were also different.

**Figure 3:**
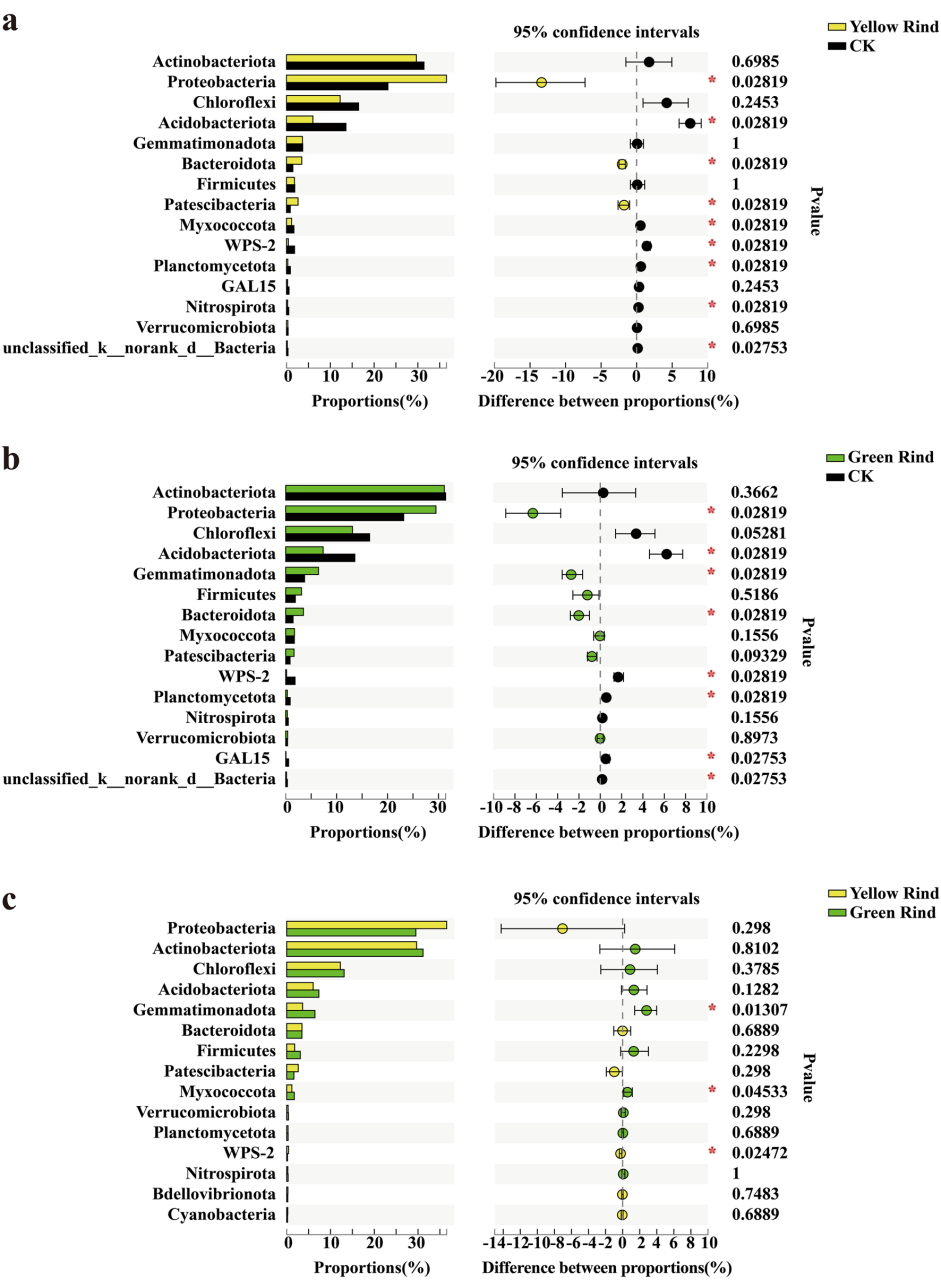
Difference test of soil bacteria between each group among yellow, green and CK at phylum level. *0.01 < *p *≦ 0.05, **0.001 < *p *≦ 0.01, ***p ≦ 0.001. (Sequences that could not be classified into any known group were assigned as “unclassified”. Some intermediate ranks in the taxonomic spectrum appeared in the comparison database without scientific names and were assigned as “norank”. That was, this rank was not named.)

**Figure 4:**
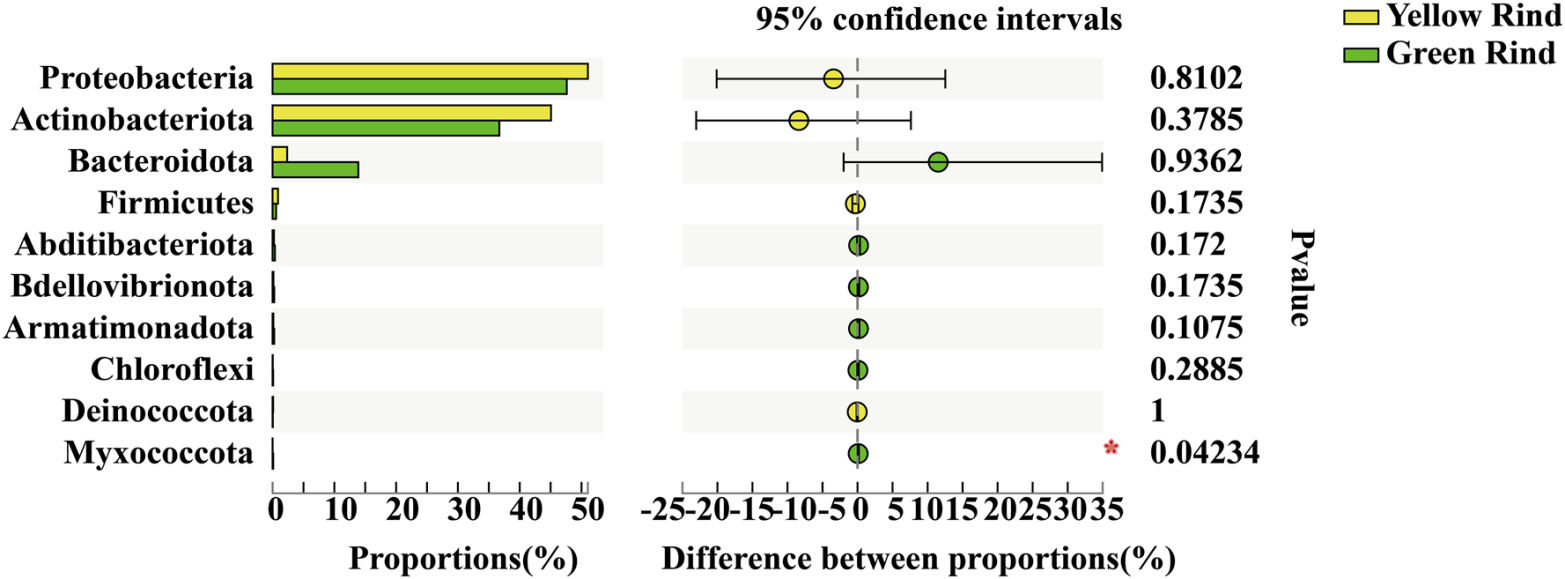
Difference test of endophytic bacteria between yellow and green rind watermelons at phylum level. *0.01 < *p *≦ 0.05, **0.001 < *p *≦ 0.01, ***p ≦ 0.001.

The Wilcoxon rank-sum test was used to analyze the significant differences at phylum level for the top 15 soil bacteria in terms of relative abundance percentage. As seen in Fig. 3, Proteobacteria, Acidobacteriota, Bacteroidota, Patescibacteria, Myxococcota, WPS-2, Planctomycetota, Nitrospirota and unclassified_k_norank_d__Bacteria were significantly different in soils between yellow rind watermelons and CK (Fig. 3a). Proteobacteria, Acidobacteriota, Gemmatimonadota, Bacteroidota, WPS-2, Planctomycetota, GAL15 and unclassified_k__norank_d__Bacteria were significantly different in soils between green rind watermelons and CK (Fig. 3b), too. Gemmatimonadota, Myxococcota and WPS-2 were significantly different in rhizospheres between yellow and green rind watermelons (Fig. 3c). Meanwhile, Myxococcota, a phylum of endophytic bacteria between yellow and green rind watermelons was also significantly different too (Fig. 4) (Wilcoxon rank-sum test, *p *< 0.05, *p *< 0.01).

**Figure 5:**
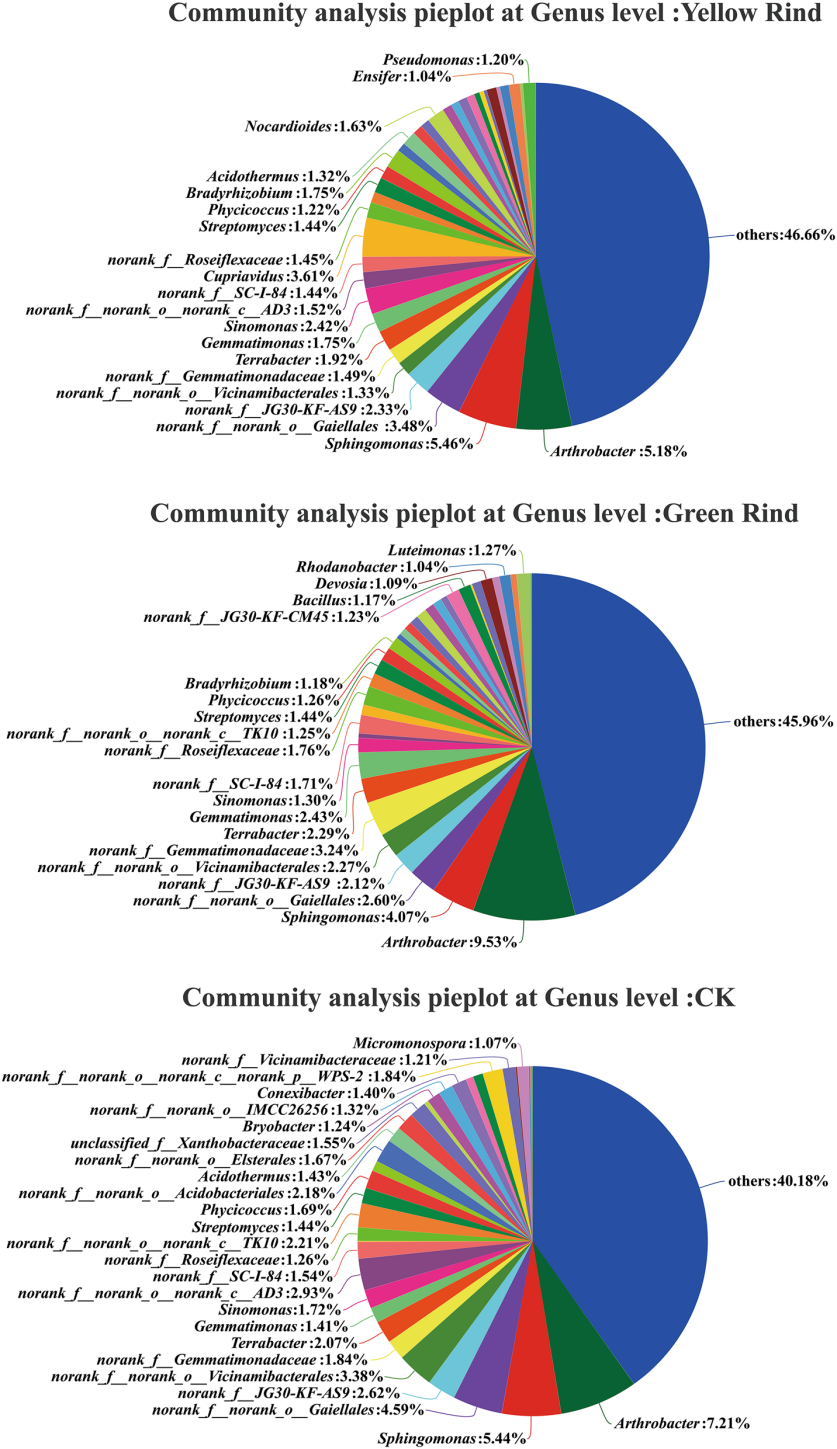
Compositions and proportions of soil dominant bacteria in rhizospheres of yellow, green rind watermelons and CK at genus level. (Sequences that could not be classified into any known group were assigned as “unclassified”. Genera making up less than 1% of total composition in each sample were classified as “other”).

The compositions and relative proportions of the soil dominant bacterial genera in CK and rhizospheres of yellow and green rind watermelons were shown in Fig. 5, respectively. As can be seen in Fig. 5, the numbers of identified bacterial genera in Yellow, Green Rinds and CK were 20, 20 and 25, respectively. Moreover, *Cupriavidus*, *Nocardioides*, *Ensifer* and *Pseudomonas* were the unique soil dominant bacterial genera in rhizosphere of yellow rind watermelon. On the contrary, *norank_f__JG30-KF-CM45*, *Bacillus*, *Devosia*, *Rhodanobacter* and *Luteimonas* were the special soil dominant bacterial genera in rhizosphere of green rind watermelon (Fig. 5).

**Figure 6:**
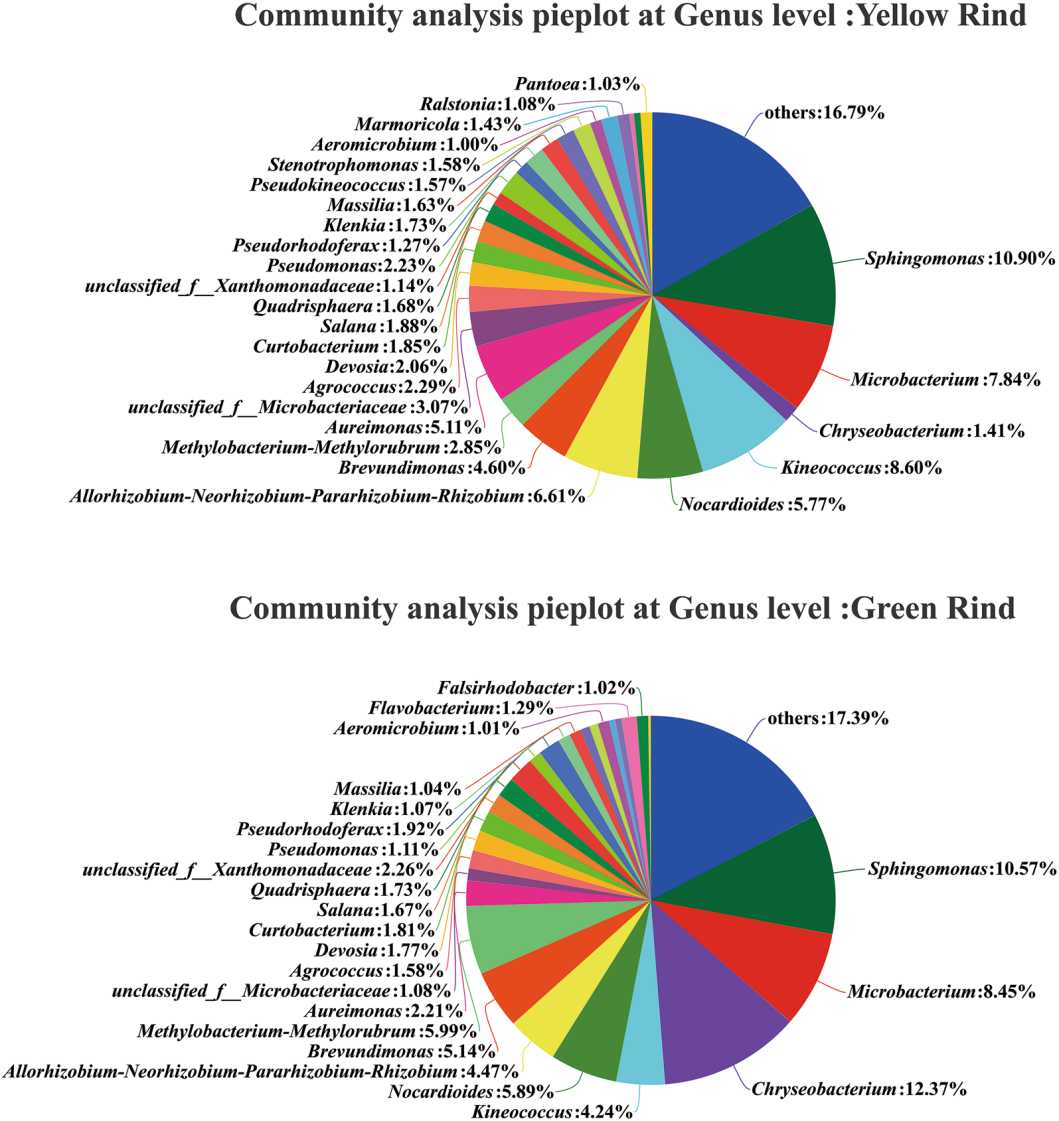
Compositions and proportions of endophytic dominant bacteria in stems of yellow and green watermelons at genus level. (Sequences that could not be classified into any known group were assigned as “unclassified”. Genera making up less than 1% of total composition in each sample were classified as “other”.)

In addition, the numbers of the dominant endophytic bacteria at genus level between yellow and green rind watermelons were 26 and 23, respectively (Fig. 6). Among them, *Pseudokineococcus*, *Stenotrophomonas*, *Marmoricola*, *Ralstonia* and *Pantoea* were the unique dominant endophytic bacteria in stem of yellow rind watermelon. By contrast *Flavobacterium* and *Falsirhodobacter* were the special dominant endophytic bacteria in stem of green rind watermelon (Fig. 6).

**Figure 7:**
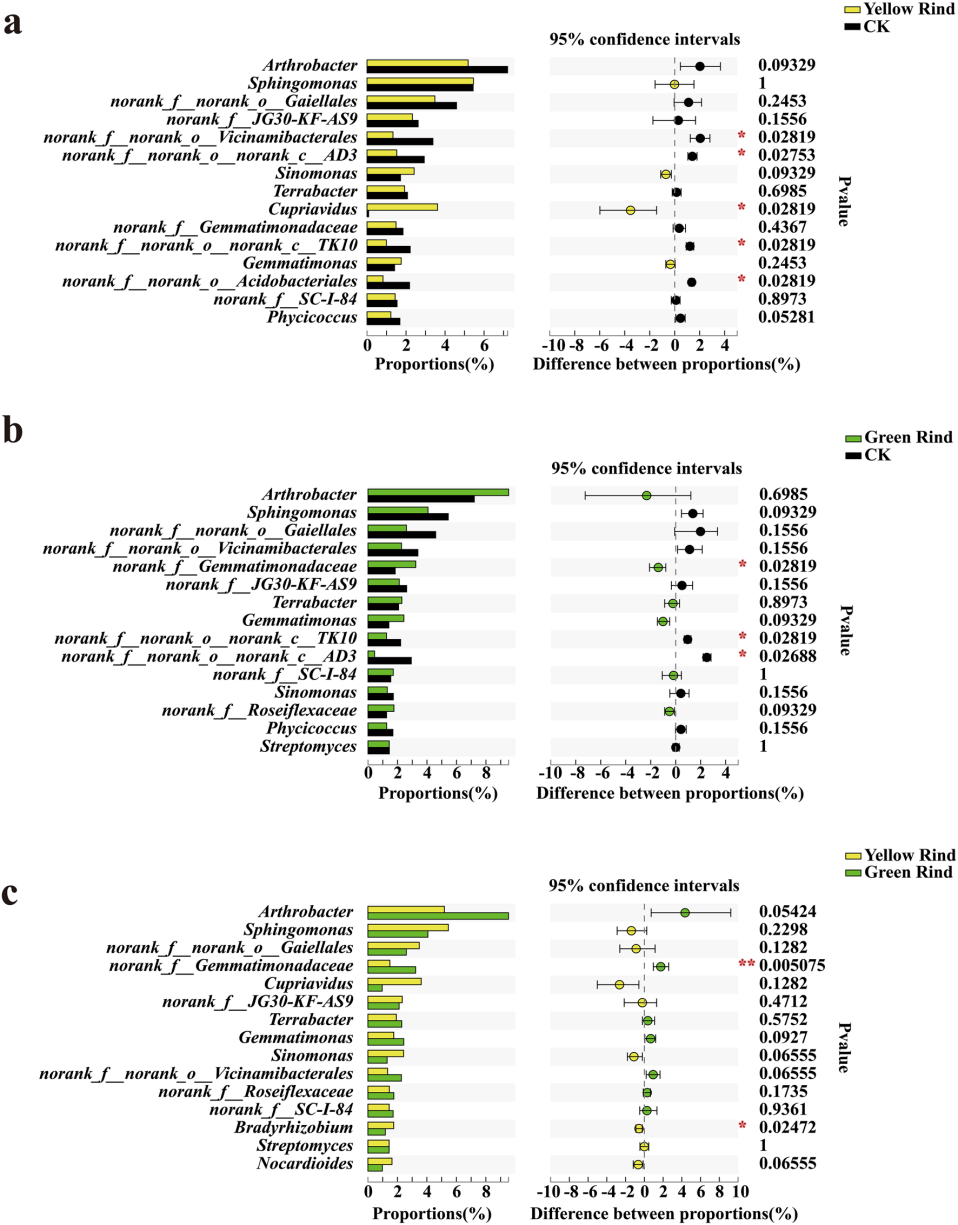
Difference test of soil bacteria at genus level among yellow, green rind watermelons and CK. *0.01 < *p *≦ 0.05, **0.001 < *p *≦ 0.01, ***p ≦ 0.001. (Some intermediate ranks in the taxonomic spectrum appeared in the comparison database without scientific names and were assigned as “norank”. That was, this rank was not named.)

The Wilcoxon rank-sum test was used to analyze the significant differences at genus level for the top 15 soil bacteria in terms of relative abundance percentage. As seen in Fig. 7. *norank_f__norank_o__Vicinamibacterales*, *norank_f__norank_o__norank_c__AD3*, *Cupriavidus*, *norank_f__norank_o__norank_c__TK10* and *norank_f__norank_o__Acidobacteriales* were significantly different in soils between yellow rind watermelons and CK (Fig. 7a). *norank_f__Gemmatimonadaceae*, *norank_f__norank_o__norank_c__TK10* and *norank_f__norank_o__norank_c__AD3* were significantly different in soils between green rind watermelons and CK (Fig. 7b). *norank_f_Gemmatimonadaceae* and *Bradyrhizobium* were significantly different in rhizosphere between yellow and green rind watermelons (Fig. 7c) (Wilcoxon rank-sum test, *p *< 0.05, *p *< 0.01).

**Figure 8:**
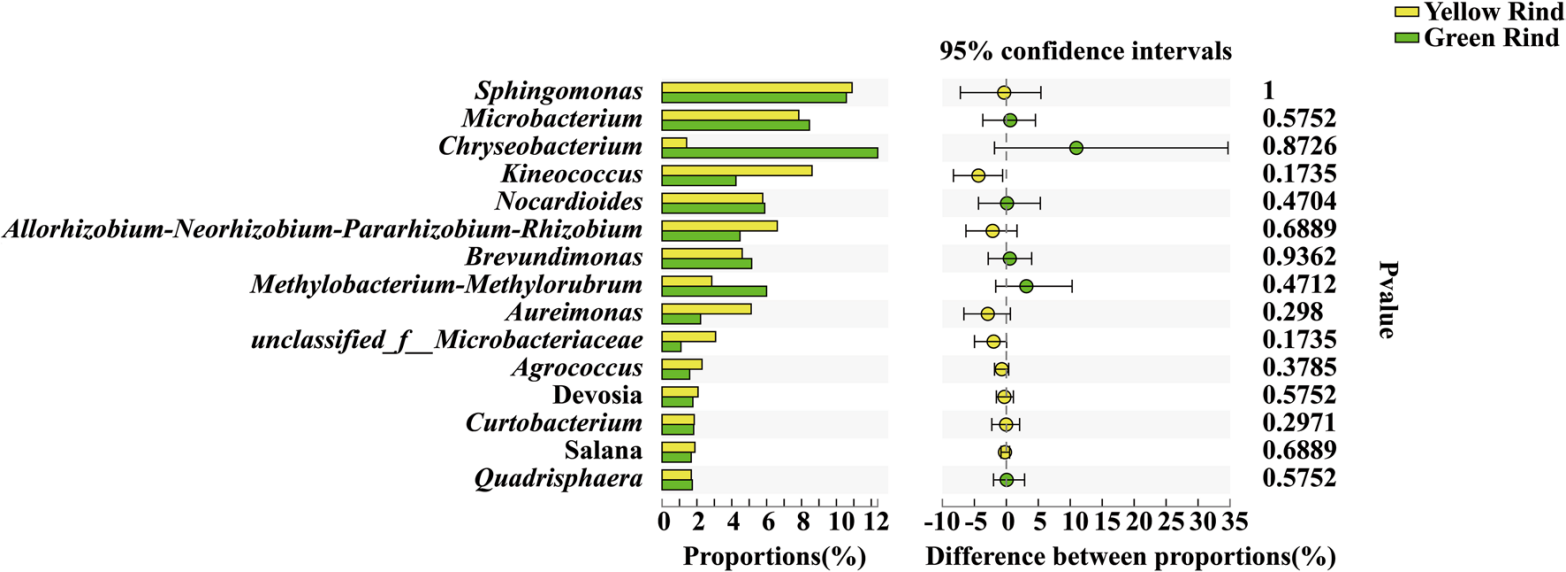
Difference test of endophytic bacteria at genus level between yellow and green rind watermelons. *0.01 < *p *≦ 0.05, **0.001 < *p *≦ 0.01, ***p ≦ 0.001. (Sequences that could not be classified into any known group were assigned as “unclassified”.)

However, there was no significant difference of endophytic bacteria at genus level between yellow and green rind watermelons (Fig. 8) (Wilcoxon rank-sum test, *p *< 0.05, *p *< 0.01).

**Figure 9:**
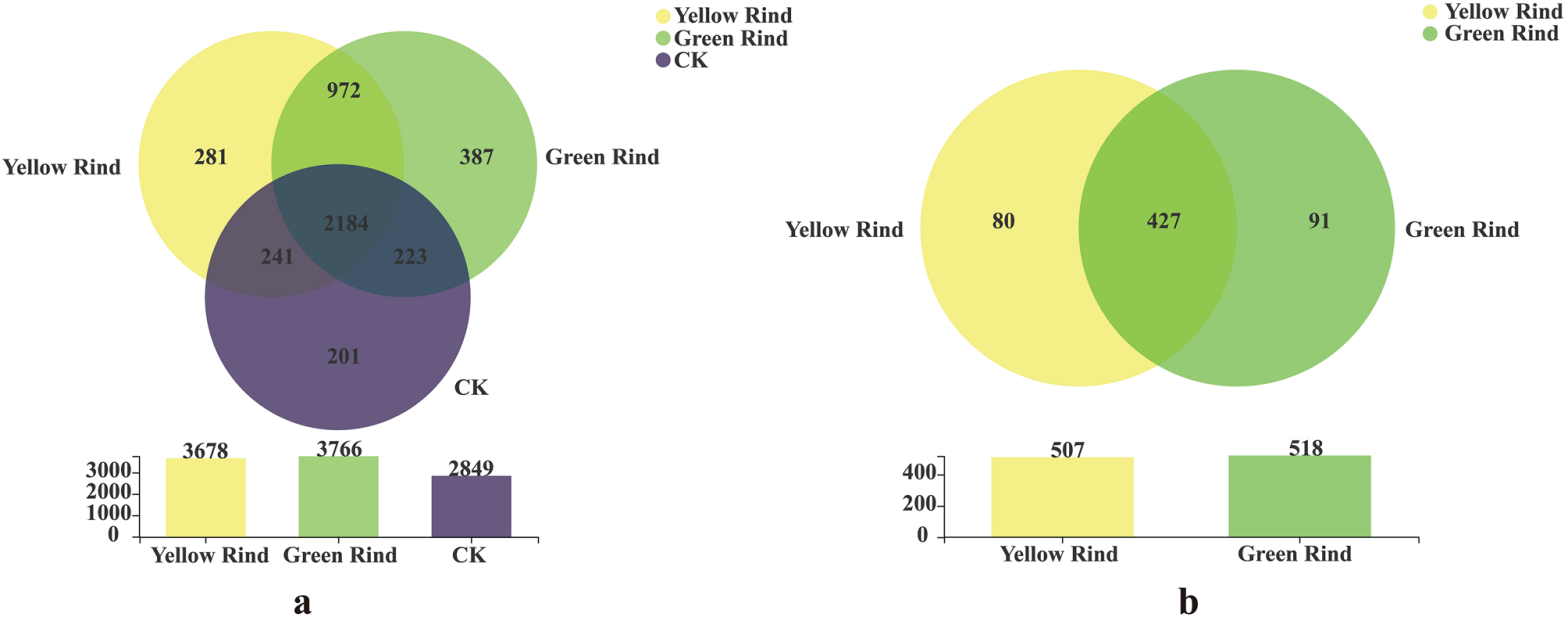
Venn diagrams of the soil dominant bacteria in rhizosphere (**a**) and endophytic (**b**) bacteria of yellow and green rind watermelons at OTU level.

Meanwhile, according to the Venn diagram at OTU level, 2849, 3678 and 3766 soil bacterial OTUs could be obtained in CK and rhizospheres of yellow and green rind watermelons, respectively. Among them, 972 common soil bacterial OTUs could be detected in rhizospheres between yellow and green rind watermelons. And 281, 387 and 201 unique soil bacterial OTUs could be detected in rhizospheres of yellow, green rinds watermelons and CK, respectively (Fig. 9a).

In addition, 427 common endophytic bacterial OTUs could be found between yellow and green rind watermelons. And 80 and 91 special endophytic bacterial OTUs could be detected in stems between yellow and green rind watermelons, respectively (Fig. 9b).

### Function predictions of rhizospheric and endophytic bacterial compositions across samples

**Figure 10:**
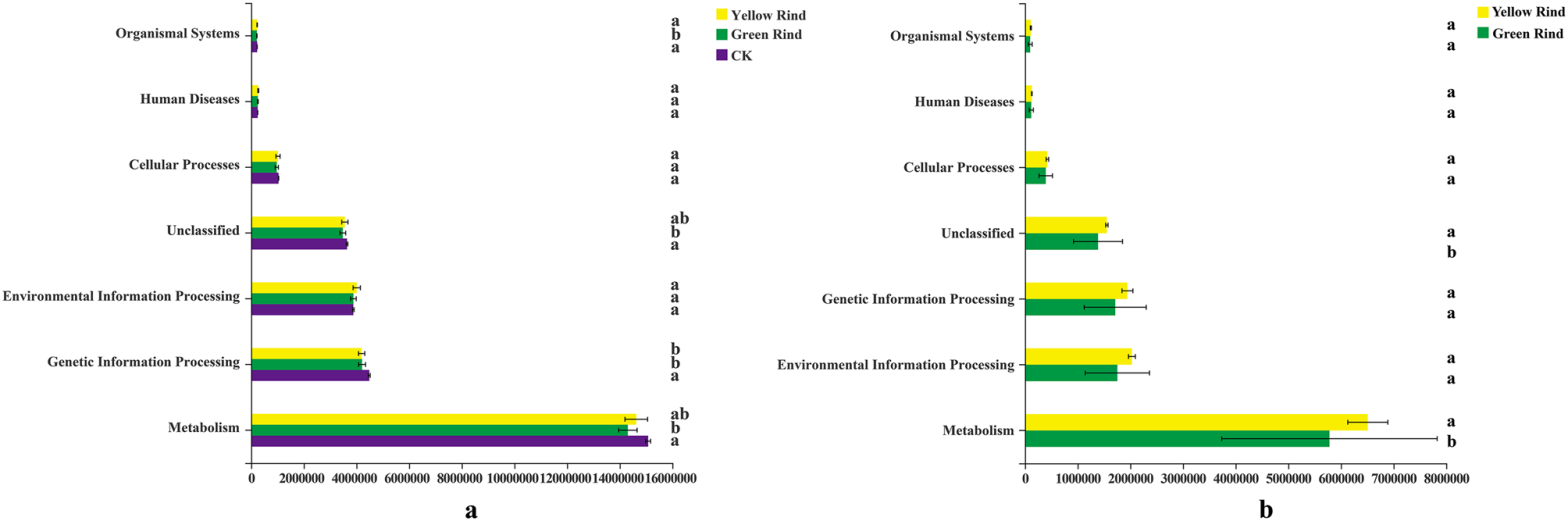
KEGG functional classification statistics of soil dominant bacteria in rhizosphere and endophytic bacteria between yellow and green rinds of watermelons (Pathway level 1). (**a**) KEGG functional classification statistics of soil dominant bacteria in rhizospheres of yellow, green rind watermelons and CK. (**b**) KEGG functional classification statistics of endophytic dominant bacteria in stems of yellow and green rind watermelons. Different letters in the same column indicate significant differences between treatments at *p* < 0.05 among the means of the different treatments. The horizontal coordinate indicates the abundance value and the vertical coordinate indicates the KEGG functional classification name.

The Phylogenetic Investigation of Communities by Reconstruction of Unobserved States (PICRUSt) algorithm was employed to predict bacterial functions in the three groups. Among them, only the pathway which related to organismal systems of soil bacteria in rhizosphere of yellow rind watermelon could be found significantly higher than those of green rind watermelon (Fig. 10a). In addition, the pathways which related to metabolism and Unclassified of endophytic bacteria in stems of yellow rind watermelon were also detected significantly higher than those of green rind watermelon too (Fig. 10b).

## Discussion

As is an important horticultural crop all over the world, watermelon rind colors are mainly shown at dark green or light green, light green-gray or yellow^3–5^. The inheritance of the watermelon rind color is a qualitative trait, but the genetic pattern and developmental mechanisms are complex^5,35,37^. In general, the color of leaves and fruit rinds are mainly depended on the contents and the formation of pigments^38^. As is well-known, chlorophyll and carotenoid are the main pigments which affecting watermelon rind color^1^. Among them, chlorophyll content is the primary factor for the green rind in Cucurbitaceae, the dark green rind watermelon contains a much higher level of chlorophyll than that of the light green rind watermelon^39,40^. It is well documented that chlorophyll degradation is positively correlated with ethylene signaling^41^. A recent study by Han et al. found that the key gene responsible for apple peel greening is the ethylene-inducible factor ERF17. Furthermore, the number of serine repeats in ERF17 affects the binding activity of ERF17 to the promoter sequences of chlorophyll degradation-related genes, and an increase in the number of serine repeats enhances the activity of chlorophyll degradation-related enzymes, and the expression of ERF17 was positively correlated with PPH (pheophytin pheophorbide hydro-lase)^42^.

In addition, carotenoids are synthesized via the isoprenoid pathway, a very large secondary metabolic pathway that synthesizes chlorophyll, gibberellin, abscisic acid, and cytokinins in addition to carotenoids^43,44^. Li et al. found that exogenous auxin delayed tomato color change mainly by inhibiting the expression of major genes in the *β*-carotene metabolic pathway^45^. Wisutiamonkul et al. also found that the ethylene inhibitor 1-MCP (1-methylcyclopropene) inhibited the production of carotenoids and delayed fruit color change by suppressing the expression of genes such as ZDS (zeta-carotene desaturase), LCY-B (lycopene beta-cyclase) and BCH (beta-carotene hydroxylase) through the inhibition of endogenous ethylene production^46^. Liu et al. had found a dramatic reduction in chlorophyll a and chlorophyll b and an increase in carotenoids in yellow rind watermelon^1^. Moreover, Zhu et al. also found that ethylene accelerated carotenoid accumulation by up-regulating the expression of carotenoid biosynthetic genes^47^. In this paper, we found that *norank_f_Gemmatimonadaceae* and *Bradyrhizobium* were significantly different in rhizospheres between yellow and green rind watermelons. As *Bradyrhizobium*, was reported for producing indole-3-acetic acid (IAA)^48^. Boiero et al. also confirmed that *Bradyrhizobium* could increase the content of plant endogenous ethylene^49^. It indicated that the enrichment of *Bradyrhizobium* in rhizosphere of yellow rind watermelon could induce higher contents of carotenoid formation. Meanwhile, we also found that *Cupriavidus*, *Nocardioides*, *Ensifer* and *Pseudomonas* were the unique soil dominant bacteria in rhizosphere of yellow rind watermelons. Among them, *Pseudomonas* can produce salicylic acid (SA) as a siderophore^50^. Previous study also confirmed that the application salicylic acid (SA), total carotenoid content, size of xanthophyll pool, and de-epoxidation rate increased significantly with an increase in SA concentration in both plant species^51^. Moreover, *Pseudokineococcus*, *Stenotrophomonas*, *Marmoricola*, *Ralstonia* and *Pantoea* were the special dominant endophytic bacteria in stem of yellow rind watermelons. *Flavobacterium* and *Falsirhodobacter* were the unique dominant endophytic bacteria in stem of green rind watermelons. However, their functions relate to rind color formation between yellow and green rind watermelons are still unknown, further researches need to be conducted.

PICRUSt function prediction analysis method has been widely applied in the study of bacterial functions in many plants^52^. Even though many soil bacterial functions are similar in rhizosphere between yellow and green rind watermelons, such as Cellular processes, Environmental information processing, Genetic information processing, human diseases, Metabolism and Unclassified. However, the organismal systems of soil bacteria in rhizosphere of yellow rind watermelons were significantly higher than those of green rind watermelons. It indicated that soil bacteria in rhizospheres of yellow and green rind watermelons were carried out different functions. In addition, similarity, some PICRUSt functions of endophytic bacteria were also quite similar between yellow and green rind watermelons, such as Cellular processes, Environmental information processing, Genetic information processing, Human diseases, Organismal systems. However, the metabolism and unclassified functions of endophytic bacteria in stems of yellow rind watermelons were significantly higher than those of green rind watermelon. It also suggested that different functions of the endophytic bacteria enriched in different phenotype watermelons (yellow and green rind watermelons). From the discussion above, it may conclude that differences of rhizospheric and endophytic bacteria are exactly recruited in different phenotype watermelons. Moreover, different functions of soil bacteria in rhizosphere and endophytic bacteria in stems of watermelons, also can be speculated relating to different rind colors formation.

In conclusion, a field experiment was carried out to elucidate the compositions of soil bacteria in rhizosphere and endophytic bacteria in stems of yellow and green rind watermelons. The results revealed that Gemmatimonadota, Myxococcota, WPS-2, *norank_f_Gemmatimonadaceae* and *Bradyrhizobium* enriched in rhizosphere of green rind watermelon; by contrast, *Cupriavidus*, *Nocardioides*, *Ensifer* and *Pseudomonas* enriched in rhizosphere of yellow rind watermelon. In addition, in comparison to the green rind watermelons, *Pseudokineococcus*, *Stenotrophomonas*, *Marmoricola*, *Ralstonia* and *Pantoea* were the special dominant endophytic bacterial genera in stems of yellow rind watermelons. Meanwhile, the organismal systems of soil bacteria in rhizosphere and the metabolism and unclassified functions of endophytic bacteria in stems of yellow rind watermelons were all significantly higher than those of green rind watermelons. All above results suggested that differences of rhizospheric and endophytic bacteria are exactly recruited as “workers” by different watermelon phenotypes relating to rind color formations.

## Methods

### Study sites description

The experiment was conducted in the experimental base of Suxu town (108° 6′ 11″ E, 22° 28′ 28″ N), Nanning city, Guangxi Zhuang Autonomous Region, southwest of China. The physical and chemical properties of the soil in field of the experimental base are as follows: pH 4.45, the contents of organic matter, total nitrogen, available phosphorus and potassium contents were 9.96 g kg^−1^, 0.74 g kg^−1^, 17.0 mg kg^−1^ and 78.0 mg kg^−1^, respectively.

### Test materials

**Figure 11:**
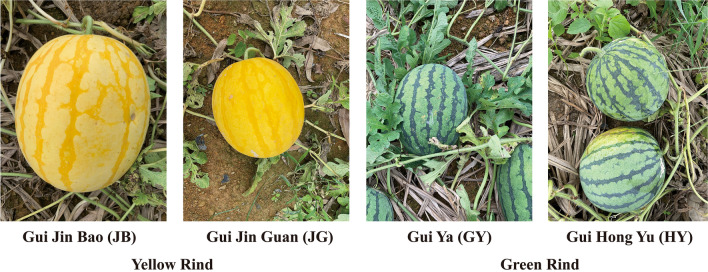
The appearance and morphological characteristics of different watermelon varieties.

The yellow rind watermelon varieties, Gui Jin Bao (JB), Gui Jin Guan (JG) and the green rind watermelon varieties Gui Ya (GY), Gui Hong Yu (HY), which all provided by Horticultural Research Institute, Guangxi Academy of Agricultural Sciences (Fig. 11) were used in this experiment for analysis. They were all planted in the same time and grew in the same filed at February, 2021 under identical management.

### Soil and plant samples collection

Soil and plant samples were collected at May 20, 2021 Rhizosphere soils from every watermelon variety were collected randomly according to the shake method described by Riley and Barber^53^. Briefly, a circle with a radius of about 25 cm was shoveled loose with a sterilized shovel at the center of the plant, the whole watermelon plant was pulled out by hand holding the base of stem. The soil attached to the roots was forcefully shaken off and carefully collected as the rhizosphere soil samples. And the three replicated soil samples from no watermelon growing area were also collected randomly as background (CK). The soil samples were passed through a 2-mm sieve and stored at − 80 ℃ for DNA extraction. Meanwhile, stem samples were collected separately from the plant using sterilized scissors and placed in sealed sterile bags and labeled. i.e., soil and appendages were rinsed from the surface of the samples with sterile water and blotted dry with sterile filter paper. The stems were placed in sterile bags and stored at − 80 ℃ for endophytic bacterial compositions analysis.

### Analysis of rhizosphere soil and endophytic bacterial compositions

The extraction, PCR amplification and sequencing of the total DNA from stem and soil samples were all completed by Majorbio Bio-Pharm Technology Co. Ltd. (Shanghai, China). The specific sequencing types and primer sequences were shown in Table 1.

**Table 1:** The name of sequence type and primer sequence.

Source	Primer name	Primer sequence	Sequencing platform
Soil bacteria	338F	5′-ACTCCTACGGGAGGCAGCAG-3′	PE300
	806R	5′-GGACTACHVGGGTWTCTAAT-3′	
Endophytic bacteria	799F	5'-AACMGGATTAGATACCCKG-3'	PE250
	1192R	5'-ACGGGCGGTGTGTRC-3'	
	799F	5'-AACMGGATTAGATACCCKG-3'	
	1193R	5'-ACGTCATCCCCACCTTCC-3'	

Microbial community genomic DNA was extracted from samples using the E.Z.N.A.^®^ DNA Kit (Omega Bio-tek, Norcross, GA, U.S.) according to the manufacturer’s instructions. The DNA extract was checked on a 1% agarose gel, and the DNA concentrations and purity were determined with a NanoDrop 2000 UV–Vis spectrophotometer (Thermo Scientific, Wilmington, USA). PCR amplification and sequencing of the total DNA extracted from the plant stem samples were performed by Shanghai Majorbio Bio-pharm Technology Co., Ltd, the hypervariable region V3–V4 of the bacterial 16S rRNA gene were amplified with primer pairs 338F and 806R, the primers 799F and 1192R were selected for the first round of PCR amplification of the V5–V7 variable region, and the primers 799F and 1193R were selected for the second round of PCR amplification of the V5-V7 variable region. PCR amplification was performed on an ABI GeneAmp^®^ 9700 PCR thermocycler (ABI, CA, USA), and the PCR products were recovered using 2% agar-gel electrophoresis. The products were purified by using an AxyPrep DNA Gel Extraction Kit (Axygen, USA) and quantified using a Quantus Fluorometer (Promega, USA)^54,55^. The purified amplicons were pooled in equimolar quantities and were paired-end sequenced (2 × 300) on the Illumina MiSeq platform (Illumina, San Diego, USA) according to the standard protocols of the Majorbio Bio-Pharm Technology Co. Ltd. (Shanghai, China). Raw reads were deposited in the NCBI Sequence Read Archive (SRA) database. (Accession Number: SRP341161).

The raw 16S rRNA gene sequencing reads were demultiplexed, quality-filtered by Trimmomatic and merged by FLASH with the following criteria: (1) the 300 bp reads were truncated at any site receiving an average quality score of < 20 over a 50 bp sliding window, and the truncated reads shorter than 50 bp were discarded, reads containing ambiguous characters were also discarded; (2) only overlapping sequences longer than 10 bp were assembled according to their overlapped sequence. The maximum mismatch ratio of overlap region is 0.2. Reads that could not be assembled were discarded; (3) Samples were distinguished according to the barcode and primers, and the sequence direction was adjusted, exact barcode matching, 2 nucleotide mismatch in primer matching^56^.

Operational taxonomic units (OTUs) with 97% similarity cutoff were clustered using UPARSE (version 7.1, http://drive5.com/uparse/), and chimeric sequences were identified and removed. The taxonomy of each OTU representative sequence was analyzed by RDP Classifier (http://rdp.cme.msu.edu/) against the 16S rRNA database using confidence threshold of 0.7^57^.

The R package was used to visualize interactions across bacterial communities in various samples, and Principal Component Analysis (PCA) was performed using unweighted UniFrac distance metrics. Meanwhile, to evaluate the bacterial composition between samples, Partial Least Squares Aiscriminant Analysis (PLS-DA) and Analysis of Similarities (ANOSIM) were also used. In addition, PICRUSt was used to estimate the functional components of bacterial communities using the Kyoto Encyclopedia of Genes and Genomes (KEGG) dataset^58,59^.

### Statistical analyses

The experimental data were analyzed using Excel 2019 and IBM SPSS Statistics 21. And the results are shown as means with their standard deviations (mean ± SD). Online data analysis was conducted by using the free online platform of the Majorbio Cloud Platform (www.majorbio.com) of the Majorbio Bio-Pharm Technology Co. Ltd. (Shanghai, China).
